# The Eighth Periodical International Ophthalmological Congress

**Published:** 1895-02

**Authors:** Alvin A. Hubbell

**Affiliations:** Buffalo, N. Y.


### THE EIGHTH PERIODICAL INTERNATIONAL OPHTHALMOLOGICAL CONGRESS, HELD AT EDINBURGH, AUGUST 7–11, 1894.

By ALVIN A. HUBBELL, M. D., Buffalo, N. Y.

(*Continued from page 356.*)

### INJURIES.

Leber (4) had found that injuries of the eye from copper or brass are generally aseptic. Test cultures, however, should be made, and if microörganisms can be grown from the extracted foreign body or from the discharge from the wound, the eye should be enucleated ; but if aseptic, the wound soon heals, and there is very little danger of sympathetic ophthalmia, as the latter is almost always septic in origin. It had not occurred in any of his cases, forty-six in number. Conservative treatment is especially indicated as long as the function of the retina is intact. He believed that in working people the retention of the globe is desired, as they consider a globe, however disfigured, better than any substitute.

Knapp reported a case in which, twenty-three years ago, he had extracted a piece of gun-cap from the bottom of the vitreus which had entered the eye through the ciliary body three days before. He did this by means of a curved, grooved hook, passed through an opening made in the sclera, the fundus of the eye being illuminated during the operation by a head-mirror. A cyst afterward developed at the point of entrance of the foreign body, which he radically removed. He had seen the patient the past winter, and found the eye in good condition and with good sight.

Roosa, of New York, asked Leber if the situation of the wound had been fully considered by him, in speaking of the danger of sympathetic inflammation. It seemed to him that even an aseptic wound in the ciliary region might cause sympathetic inflammation, while one in the cornea or sclera might not.

Noyes had seen cases in which pieces of gun-cap remained visible on the retina for many years without further injury ; but it had generally been his rule to enucleate eyes thus injured. It would be a great relief, both to him and his patients, if Prof. Leber could show him how to save such eyes, for the saving of even a deformed eye is a most precious attainment.

Kipp, of Newark, N. J., mentioned a case in which a piece of gun-cap entered the eye through the cornea and lens and lodged in the lower half of the retina, twenty-four years ago. The lens became absorbed, when the retina was seen to be clear, except at the point where the foreign body was lodged. There was no detachment, and vision is $\frac{20}{80}$.

Meyer, of Paris, had published a curious case, in which a piece of gun-cap had entered the eye of a boy and could be seen resting upon the supero-external part of the fundus. A year later it was found to have been spontaneously expelled at the point of entrance, in the midst of a small subconjunctival abscess.

Leber, in closing, said that not in one case of his had he observed either sympathetic irritation or inflammation. He did not fear sympathetic irritation by reflex action, because this could be inhibited by performing enucleation afterward, if necessary. In two cases in which he had, for greater security, enucleated the eyeball, anatomical examination gave him the impression that the enucleation might have been avoided.

Panas (5) concluded his paper with regard to traumatic motor paralysis of the eye due to compression, as follows:

1. In the majority of cases the paralysis is due to fracture of the base of the skull.

2. The absence of depression of the bones of the vault of the skull is no proof that fissures do not exist in the base.

3. The nerves most frequently paralyzed are the sixth pair, owing to their being most intimately connected with the bone in question.

4. The compression may be caused either by the fracture itself or by hemorrhage into the cranium. In the former case, the paralysis is more or less immediate; in the latter, it may not appear until late, as in plastic exudations by phlogosis.

Chibret had recently had a case in which a lad had had his head squeezed transversely, through some mismanagement of an elevator, and three months after the accident had paralysis of both sixth nerves. He was obliged to practise a tenotomy of both internal recti muscles to enable the patient to walk without difficulty.

Dufour (18) read an interesting paper on the accidental retro-choroidal hemorrhages which sometimes take place after operations on the eye. The accident was rare, Alb. von Graefe having only met with it twice in his extensive experience. The origin of the hemorrhage is retro-choroidal, as was obvious from a case in which he had seen it supervene after an iridectomy for the relief of extreme pain in a case of absolute glaucoma. The eye was enucleated, and examination showed that a large hemorrhagic focus had pushed the retina and choroid toward the wound.

No examination of the eyes or of the heart and vessels had ever enabled him to diagnosticate, beforehand, a liability to hemorrhage. At first he had resorted to hemostatic measures, such as refrigeration, pressure and derivation, without success. In one case he enucleated the eye. During the past four years, as soon as he had noticed symptoms of this complication, he had injected a large dose of morphine into the temple, near the eye. He suggested adding to the injection a substance susceptible of producing nausea. He has treated three cases in this manner with much less serious results than by other methods.

Ayres, of Cincinnati, reported a case in which this accident occurred four hours after iridectomy for glaucoma in a patient sixty-five years old. The entire vitreus was forced out. The eye was immediately enucleated.

Gruening, of New York, had seen the accident follow a cataract extraction in a man nearly ninety years old, in his own practice, and in another patient in Paris almost eighty years old.

Power, of London, thought it was remarkable that the accident was not more frequent, in view of the sudden reduction of tension in operation. He had had two or three cases occur in eyes that were not healthy. In one case an iridectomy had been performed, and in another a cataract extracted.

Darier thought the idea of provoking nausea to arrest hemorrhage seems practical, and suggested the injection of a small dose of apomorphine.

Dufour said, in closing, that the first two cataract patients were seventy and eighty years old respectively ; the two glaucomatous cases were over sixty, and the last three operated cases were nearly seventy. He thought a solution of apomorphine and morphine would suit very well. But the cases occurred unawares, when the apomorphine was not immediately at hand. It would be well to have it in reserve.

MEDICAL THERAPEUTICS.

Mules (6 *b*) reported unusually good results in the treatment of corneal ulcers by placing an antiseptic wafer over the cornea and then using an iodoform pad with a bandage over the eyes for several days. The wafer is made of gelatin, a saturated solution of boracic acid and iodoform in impalpable powder. It is dipped in boracic acid solution before being used. Cicatrization often occurs in three days.

Noyes said that Dr. Williams, of Cincinnati, at the meeting of this congress in 1876, recommended the use of pure carbolic acid to corneal ulcers, and he (Noyes) had many times proved the value of this procedure. Only one application is generally necessary, the result and the indications being practically the same as those in the use of the actual cautery.

Vian (20) recommended crude petroleum in the treatment of diphtheritic conjunctivitis, and reported three cases illustrating its effect. The first was one of ophthalmia neonatorum, which in a few days was transformed into pseudo-membranous conjunctivitis. After failure with various remedies and loss of the right cornea by necrosis, he began cleansing the mucosa and palpebral folds twice a day at first, with a pencil dipped in petroleum, and later every three hours. Improvement began at once and recovery rapidly took place.

The second case was one of marked diphtheritic conjunctivitis in a child three years old, a grayish exudate completely covering the conjunctiva, the lids being indurated and swelled and the cornea becoming cloudy. The case was fully recovered after forty-six days under the applications of the petroleum.

The third case was in a boy three years old, but the disease was less severe. He was restored in a month by the same treatment.

He suggests that the applications of the petroleum should be made every hour.

Bach (24) believed that sub-conjunctival injections were ineffectual in corneal abscess, and was sustained in this belief by experiments made on rabbits in which he had produced corneal ulcers and abscesses by staphylococci. He preferred cauterization to grattage of the abscess as more certain and also as less dangerous. Hypopyon implies an inflammatory complication on the part of the iris and ciliary body.

Chibret (29) for twenty-nine years had suspected that iodide of potassium did not possess specific value in syphilis. Nineteen years of ophthalmic practice had confirmed him in this opinion.

Observation and experience had established in his mind the following propositions:

1. In ocular syphilis, mercury, singly, acts nearly always; iodide of potassium, singly, never acts.

2. In general syphilis, mercury, alone, acts nearly always, and on all the accidents; iodide of potassium, alone, only acts on certain accidents and in an inconstant manner.

3. In ocular and general syphilis, mercury, alone, may serve as a test to fix the etiological diagnosis.

4. Mercury, alone specific in syphilis, is in the meantime a poison of the organism, especially of the nervous system; whence the gravity of nervous syphilis.

5. Iodide of potassium, as a counter poison to mercury, is frequently indicated in syphilis to eliminate or make tolerable the mercury.

6. The iodide acts, moreover, on lymphatism and on rheumatism.

7. Severe syphilis is only affected by mercury alone, or in combination with iodide.

Saturation and poisoning (salivation) by the mercury are the formal indications for the iodide of potassium.

He prefers to administer mercury by injections into the buttocks. He uses the soluble salts of mercury, and preferably the cyanide. He dissolves this in the proportion of 1 to 200 of water and glycerine with a slight addition of cocaine. A medium dose of this is one-half centigram, and this should be repeated daily for from four to nine days, and then three times a week. If diarrhea supervenes, the injections should be suspended. The treatment, with occasional interruptions, should be continued two years at least, and even three or four in subjects in the decline of life.

Darier (30) gave preference to the injection of soluble salts of mercury, especially to the cyanide, when mercurial treatment is required in ophthalmology. He employed them largely diluted, and repeated them every day or every other day, according to indications. When the injections were not practicable, he used inunctions. He sometimes used the "massive injections of insoluble salts of mercury" in cases which had already had a certain amount of mercurial treatment and were tolerant of it.

Intra-ocular injections were especially indicated in serious affections of the vitreus, deep-seated traumatic infections, advanced sympathetic ophthalmitis, grave syphilitic troubles which have resisted both general treatment and sub-conjunctival injections.

Sub-conjunctival injections have numerous indications: (1) they act as the most powerful and prompt means of practising antisepsis in traumatic and operative infection, in infective ulcers, and abscesses of the cornea with hypopyon ; (2) they have a strong, resolutive action on torpid, parenchymatous keratitis, on choroidal exudations, and on plastic iritis when no well-marked nervous stasis is present ; (3) as an antisyphilitic, their action on the ocular manifestations of syphilis in all its stages is rapid and sure.

The principal contra-indication of sub-conjunctival injections is where there exists a vascular engorgement of the pericorneal network. The resorption is then very slow, and there is often violent reaction which is more alarming than dangerous.

Gutman had tried Darier's method in fifty cases without success. In a case of severe specific irido-choroiditis, it made the disease much worse, and in another case hypopyon supervened.

Chibret had used sub-conjunctival injections of cyanide of mercury in ocular syphilis, especially in interstitial keratitis, with no question as to their efficacy. In suppurative diseases of the cornea and other diseases he had had variable results.

Hess and Bach, in experiments with these injections of corrosive sublimate in rabbits, had not found the slightest effect on the course of corneal ulceration.

Deutschmann, who had made over 2,000 sub-conjunctival injections of corrosive sublimate, showed himself a partisan to this method. He had found them useful in both specific and non-specific diseases of the eye, though more so in the former. In parenchymatous keratitis and acute iritis they are very beneficial. In corneal ulcers they are preferable to the actual cautery. These injections have the special merit of shortening morbid processes. But in post-operative suppuration they are not the best means of controlling the situation. They are also without effect in affections of the optic nerve.

Dufour affirmed the good results obtained by sub-conjunctival injections. He said it was probable that the quantity of corrosive sublimate necessary to be effective was much less than had been believed.

Darier replied that, contrary to Hess and Bach, mercury had been found in the eye by electrolysis in the experiments of Bonhi, Gallemaerts and Joly. The experiments of Hess demonstrated only that rabbits are refractory to these injections. From a clinical point of view, they have given good results to those who have used them on a large scale.

VALUDE (36) desired to add one or two recent observations to the facts he had published during the past year on the use of antipyrin in certain forms of optic-nerve atrophy, and to mention a case published by Dr. Bistis, of Constantinople, where antipyrin had succeeded in ameliorating an optic-nerve atrophy which had resisted a variety of treatment.

The antipyrin should be given by hypodermic injections, using two grams of a saturated solution (one gram to two grams of water) every two days. This treatment is not suited to all forms of optic atrophy. It is only indicated in those which are consecutive to descending neuritis and which have their origin in meningitis or pachy-meningitis, localized or general. In these cases the amblyopia has been preceded and is accompanied by headaches and there were signs of optic neuritis before the atrophy.

The treatment by antipyrin suppresses the headache and increases the acuity of vision when all other treatment has failed. The most useful increase of the visual acuity is for near work.

### SURGICAL THERAPEUTICS.

SNELLEN (1) read a paper recommending the covering of all operative and accidental wounds of the cornea and sclerotic by conjunctival flaps. In cataract extraction, Graefe's knife should be used and a conjunctival flap made. After enucleation the suturing of the conjunctiva favors healing. In wounds of the cornea or sclerotic, conjunctival flaps should be cut and stretched over them by sutures. After strabismus operations the conjunctiva should be made to cover the wound.

KNAPP (2) read a paper on 600 cases of cataract extraction, which was listened to with great interest. He said that during the last four years he had performed 630 cataract extractions, but 30 of these being so complicated that a good result was impossible, were excluded from the report. Among the 600 extractions 52, or 8.6 per cent., were combined with iridectomy, indications for the latter being: (1) pathological condition of the eyes; (2) a manifest tendency to prolapse of iris, that is to say, the impossibility of obtaining a central pupil and maintaining it in its place during the eye movements, or while carrying the patient from the operating chair to the bed, and for fifteen or twenty minutes afterward; (3) accidents occurring in the course of the operation. The visual results were good in 49, bad in three cases.

Out of the other 548 operations of simple extraction, *retention of the iris by constriction* took place in 55 cases (10.03 per cent.), a condition which was treated by operation (iridectomy, ablation with the aid of a galvano-cautery,) in 14 cases, that is to say, in 25.5 per cent. of the number of cases of incarceration of the iris, or 2.5 per cent. of the total number of extractions, among which were two cases of extractions with iridectomy. Among the 55 cases the result was good in 49 ($\frac{20}{20}$ in 7 cases), fairly good in 2, loss of eye in 2, and loss of both eyes in 2. In one of the last the eyes were degenerated, and the other presented an extremely marked rheumatic diathesis.

Among the 600 cases of extraction, he performed 403 operations for secondary cataract, that is, 67 per cent. Almost all consisted in discission with a knife needle. The reason for performing so many secondary operations was that he wished to give the patients the greatest possible visual acuity and to make it permanent. He had done this operation so often that he regarded it as almost wholly free from danger. In the present series of cases, visual acuity diminished a little only in eight or ten, but in one case the eye was completely lost in consequence of acute glaucoma, which supervened while the patient was returning to his home, which was situated at some distance from New York. The nature of the disease was not recognized, and the patient was treated by instillations of atropine. This was the second case of that kind which had come to his knowledge, but these two were the only cases of loss of the eye in at least 1,500 operations of secondary discission.

Acute glaucoma is an inherent consequence of discission; but it can invariably be cured by myotics, or iridectomy, and possibly by paracentesis of the anterior chamber. Among the 403 operations mentioned in this report, it occurred in twelve cases, or 3 per cent.; in four of these it yielded to the application of eserine; seven were healed under the influence of iridectomy, and the twelfth was the one to which he had referred as resulting in loss of the eye.

Little, of Manchester, related his experience of the past five years, which included 428 extractions, of which 322 were with iridectomy and 106 without. In the latter he had four failures, of which two were from suppuration and one from glaucoma. In ten cases, two of which were lost, as stated, there was prolapse of the iris. In seven of these the iris was excised without bad effects and in one the prolapse was not touched.

Panas spoke of the treatment of secondary cataracts (secondary opacities of the capsule), and said that he had practised the secondary operation, by extracting the capsule, for more than twenty years, without causing any primary or consecutive complications, and with the result of raising the vision from $\frac{1}{15}$ to 1. He had twice seen glaucoma after simple extraction of cataract.

Swanzy, of Dublin, said he was an advocate of the combined method, the iridectomy being small. In 200 consecutive cases he had had but four of incarceration of the iris in the wound, or 2 per cent. He would be glad to adopt the simple method when Prof. Knapp had reduced his cases of iris prolapse and incarceration to 1 per cent., or, at any rate, to less than 2 per cent. Iridectomy had, moreover, no disadvantages beyond the unimportant one of a very slight disfigurement, which neither the patient nor his friends often discover.

Noyes said that, apart from cases in which there was (1) undilability of the pupil, (2) the presence of a large amount of cortex, and (3) increased tension of the ball, he preferred the simple operation. His incision was almost a semicircle of the cornea, and the capsulotomy was in the form of an inverted T. He used atropine, both before and after the section, thus preventing adhesion of the iris to the capsule. The disadvantages of iridectomy in extraction were: (1) the additional steps added to the operation; (2) the painfulness of the operation, in most cases, of excising the iris; (3) the occurrence of incarceration of the iris or capsule in no small number of cases.

Hansen-Grut, of Copenhagen, said that they had all practised what Prof. Knapp had described. But all could not keep their patients sufficiently long to perform the discission, which he advises not to be done till three weeks or more after the extraction. Knapp seemed to have more obedient patients. On the whole, they would do well not to go far into the discussion of methods of extraction. Every experienced operator believed his own method to be superior to that of any other man, and would not be taught. The old practitioner will not benefit by discussion, and the younger operator will only be puzzled.

Critchett, of London, said he was in accord with Swanzy. To avoid the pain of iridectomy, to which Noyes had alluded, he had adopted the plan, since the first introduction of the use of cocaine, of passing, after the section is made, a few drops of a fresh, sterilized solution of cocaine directly into the anterior chamber, by means of a curette. At the end of a minute or two the iris is anesthetic, and can be excised without pain. No ill effects had ever followed. In discission of the capsule, he preferred a horizontal incision.

Teale, of Leeds, said that, having been for the past twenty years an advocate of the simple extraction, it was his conviction that, with a suitable section, such as he had described in his Bowman lecture in 1893, the simple involves no difficulties from which the combined operation is free; that it has technical advantages over the latter; that in its average results it is equal to it, and in its perfect results superior.

Snellen thought that statistics could not decide the comparative value of different methods of extraction of cataract. The choice of patients, the skill of the operator, the ability of assistants, the quality of instruments, and other circumstances must be taken into consideration. In the simple operation, he never replaces a prolapsed iris, but excises it at once. To prevent prolapse of the iris, pilocarpine should be applied during the operation, the eye must be thoroughly anesthetic, no speculum should be used, and the section should be large. The capsule may be needled two weeks after the extraction, when it is easily done.

Knapp, in replying, said that it was on the strength of his recent experience that he maintained that the simple operation is not only the safest but the best operation. Swanzy credited him with 10 per cent. of prolapses, but only fourteen cases, or 2.5 per cent. of all, had required operative treatment. The others recovered spontaneously, some with persistence of a small unirritative protrusion, not seriously interfering with sight, and in the others the prolapse disappeared. The eye, after a combined extraction, was, by the frequent incarceration of invisible iris and capsule in the scar, less resistant to obnoxious influences than when the iris was preserved. He practised secondary discission in about two-thirds of his cases, and few patients objected to it. If successful, it left an ideal eye. The discission can be done at any time, without notable reaction, if the pupil is free from inflammatory products.

(*Concluded next month.*)

